# Greater Colo-Rectal Activation Phenotype in Exercised mdx Mice

**DOI:** 10.1371/currents.md.230ed3d6559b171e10279fc16e9ebef3

**Published:** 2018-05-02

**Authors:** Marie Nearing, James Novak, Terence Partridge

**Affiliations:** Children's National Health System, Children's Research Institute, Center for Genetic Medicine Research, Washington DC, United States; Center for Genetic Medicine Research, Children's National Health System, Washington DC, United States; Center for Genetic Medicine Research, Children's Research Institute, Children's National Medical Center, Washington DC, United States

## Abstract

**Introduction:**

Duchenne Muscular Dystrophy is a genetic disease that is caused by a deficiency of dystrophin protein. Both Duchenne Muscular Dystrophy patients and dystrophic mice suffer from intestinal dysfunction.

**Methods:**

The present study arose from a chance observation of differences in fecal output of dystrophic vs. normal mice during 20­minutes of forced continuous treadmill exercise. Here, we report on the effects of exercise on fecal output in two different dystrophic mutants and their normal background control strains. All fecal materials evacuated during exercise were counted, dried and weighed.

**Results:**

Mice of both mutant dystrophic strains produced significantly more fecal material during the exercise bout than the relevant control strains.

**iscussion:**

We propose that exercise-­induced Colo-­Rectal Activation Phenotype test could be used as a simple, highly sensitive, non­invasive biomarker to determine efficacy of dystrophin replacement therapies.

## INTRODUCTION

Duchenne Muscular Dystrophy (DMD) is an X-­linked genetic disease characterized by the lack of the Dystrophin protein in the muscle tissue of affected individuals. While the predominant problem associated with DMD lies with the striated skeletal and cardiac muscles, a high percentage of DMD patients also suffer from gastrointestinal dysfunction. Constipation, gastric hypomotility, gastric dilation, and delayed gastric emptying are among the gastrointestinal motor abnormalities seen in DMD patients 1^, ^2^, ^3^, ^4.

mdx mice, the animal model of DMD, also present symptoms of digestive dysfunction. Dystrophic mice exhibit delayed intestinal transit time, decreased fecal output, and gastrointestinal contractility disturbances 5^, ^6^, ^7. These demonstrations of differences in gastrointestinal function of dystrophic vs. normal animals have employed variably invasive methods that preclude their use as routine investigational tools for serial monitoring of individual mice.

We noticed a gross difference in fecal output between the Exon52­-null mdx mouse (mdx52) and its background control C57BL/6 (BL6) strain mice when subjected to a 20­-minute forced treadmill run. To investigate whether this difference was simply (1) a strain difference, arising perhaps from genetic drift between the dystrophic and control colonies, or (2) was related to the lack of dystrophin, we subsequently repeated the comparison on the C57BL/10ScSn­-mdx (mdx), which carries a natural nonsense point mutation in exon 23, and its background control C57BL/10ScS (BL10). Dystrophin expression is eliminated in both dystrophic strains, yet numerous variations can be found between the dystrophic strains that differentially affect functional and structural outcomes^17^, such as expansion of revertant fibers^19^, expression of shorter isoforms of dystrophin in the mdx strain^20^, and abnormal electroretinograms in mdx52 similar to those in DMD patients^16^^, ^18.

Here, we present a simple protocol that arose from a chance observation from an unrelated study of the effects of routine enforced exercise on dystrophic and normal mice. This protocol is simple and sensitive enough to be potentially used as a routine measure of success in restoration of dystrophin by following variations in fecal output.

## MATERIALS AND METHODS

**Animals**

All animal procedures were approved and performed according to the Children’s National Health System’s Institutional Animal Care and Use Committee (IACUC). Animals were housed at a density of up to 5 animals per cage and maintained in pathogen-­free conditions under 12­-hour light/12-­hour dark cycles at a temperature of 18­-23°C and 40­-60% humidity. The mice were allowed ad-­libitum access to standard mouse chow and drinking water except during exercise or resting protocol periods. All animals used in the study were male mice between 1-­5 months of age, and no animals were euthanized throughout the course of these experiments. Both wild-­type strains, C57BL/10ScSn (BL10) and C57BL/6 (BL6), as well as the dystrophic C57BL/10ScSn-Dmdmdx (mdx) strain, were originally obtained from Jackson Laboratories and bred in-house at The Children's National Health System Research Animal Facility. The exon 52-deficient X-chromosome linked dystrophic mouse model generated on the C57BL/6 background (mdx52) was originally obtained from the laboratory of Dr. Shin'ichi Takeda (National Center of Neurology and Psychiatry, Japan) and bred in-house at Children's National Health System.

**Experimental Procedures**

Exercise Protocol: Normal (BL10, n = 10; BL6, n = 6) and dystrophic (mdx, n = 9; mdx52, n = 6) male mice were exercised on a treadmill (Columbus Instruments, Columbus, Ohio) at 0° incline for 20-­minutes without rest at 12 meters/minute, as previously described^8^. This protocol was conducted within the animal house within its set temperature range and during the 12 hour standard ‘daylight period’ employed in our animal unit. All animals used for the exercise protocol were studied longitudinally, following the same BL10 and mdx male mice and exercising them at 8, 9, and 20 weeks of age. Similarly, the same BL6 and mdx52 male mice were followed and exercised at 4, 8, and 12 weeks of age. Animals were continuously monitored during this exercise period; animals that drifted onto the back platform of the treadmill were pushed gently back onto the belt with a wad of tissue paper.

Resting Protocol: Normal (BL10, n = 10; BL6, n = 8;) and dystrophic (mdx, n = 8; mdx52, n = 9) mice were individually placed in plastic cages for 20-­minutes undisturbed. Animals of various ages were used to determine age effect on resting fecal output (BL10: 7, 14, 18 weeks; BL6: 8, 15 weeks; mdx: 7, 15 weeks; mdx52: 6, 11, 16 weeks).

Fecal Sample Collection: Fecal pellets excreted during the 20-­minute exercise or resting period were collected, counted, and weighed after being dried at 37°C for 10­-minutes.

All exercise and resting protocols were performed between 8:00 A.M. and 11:00 A.M. in an isolated room maintained at 21°C, 65% humidity, and <80 watts of light.

**Statistical Analyses**

All data are reported as mean values ± standard error of the mean (SEM). The consistently zero values obtained from BL6 control mice limited us to non­parametric statistical analysis. The non­parametric Spearman correlation (correlation coefficient r) was performed between both fecal output rate and weight per fecal pellet over age within the same protocol and strain. Statistical significance between strains within a protocol was assessed by the non­parametric Wilcoxon rank sum (Mann­-Whitney) test. The level of significance was set at P < 0.05.

## RESULTS

**Fecal output remains constant across all ages regardless of exercise/resting protocol or strain.**

To determine age effect on fecal output, animals of various ages were studied. No correlation was found between fecal output rate (Fig. 1A-­B; r = ­-1.00 to 0.87) or weight per fecal pellet (Fig. 1C­-D; r = -­0.80 to 0.50) over age. Correlation for exercised BL10 and BL6 animals were not calculated due to the absence of fecal output from some animals at certain time­-points. Due to the absence of age effect on fecal output, subsequent analysis was performed from pooled data across all ages.

**Fecal output remains constant across all ages regardless of exercise/resting protocol or strain. (A) Resting and (B) exercised fecal output rate over age. (C) Resting and (D) exercised weight per fecal pellet over age. All data are reported as mean values ± SEM with level of significance set at P < 0.05.**
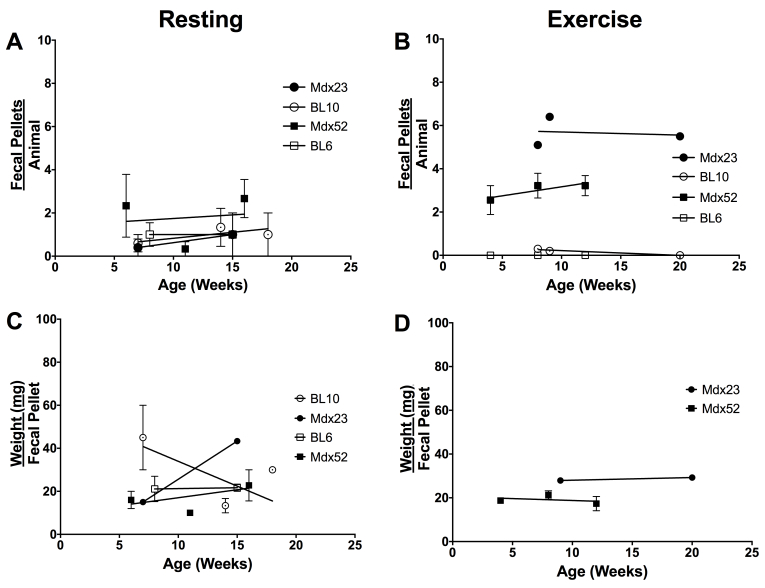

**Fecal pellet weights are constant between all strains regardless of exercise/resting protocol.**

To determine whether the altered GI function in dystrophic mice correlates with altered fecal weight, the mean fecal weight per pellet was calculated for normal and dystrophic animals within each protocol group. (Fig. 2) No significant differences in weight per fecal pellet were found between strains or protocol (Resting vs. Exercise; BL10: 29.33 ± 8.59 vs. 16.83 ± 4.075; mdx: 29.17 ± 14.17 vs. 26.48 ± 1.779; BL6: 21.33 ± 3.27 vs. No data; mdx52: 18.39 ± 4.019 vs. 19.40 ± 3.00; n = 2­6). Absence of fecal output from exercised BL6 animals precluded calculation of weight per pellet from this group.

**Fecal pellet weights are constant between all strains regardless of exercise/resting protocol. Weight per fecal pellet in resting (open circles) and exercised (closed circles) animals. Data for exercised BL6 animals are not shown due to the complete absence of fecal output. All data are reported as mean values ± SEM with level of significance set at P < 0.05.**
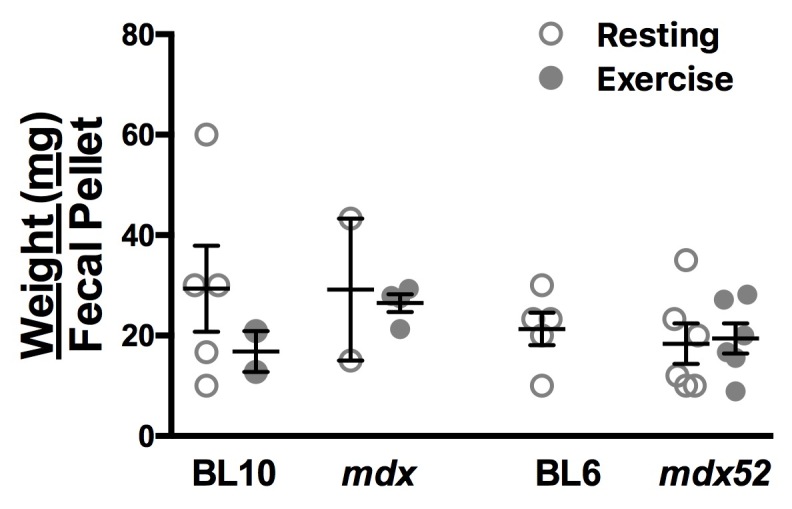

**Resting animals show comparable fecal output rates across all strains.**

To eliminate the theory that the higher fecal output in dystrophic mice is due to lower fecal output during times of rest that result in higher output during periods of exercise-­induced stress, resting baseline fecal output rate was studied in normal and dystrophic animals subjected to the resting protocol. (Fig. 3) Normal and dystrophic mice of both strains defecated at similarly low rates that were statistically indistinguishable from one another (BL10: 0.90 ± 0.35; mdx: 0.63 ± 0.42; BL6: 1.25 ± 0.45; mdx52: 1.78 ± 0.62; n = 8­10).

**Resting animals show comparable fecal output rates across all strains. Rate of fecal output in resting animals. All data are reported as mean values ± SEM with level of significance set at P < 0.05.**
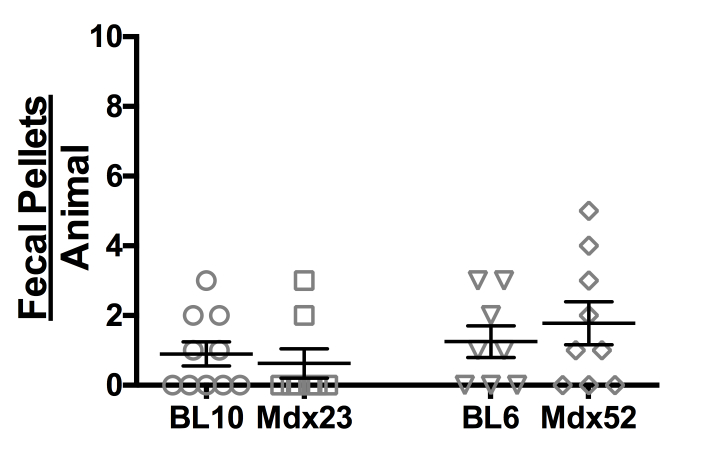

**Exercised dystrophic mice show significantly higher rates of fecal output than normal mice.**

To determine the effect of exercise-­induced stress on fecal output, fecal output rate was studied in normal and dystrophic animals subjected to the exercise protocol. (Fig. 4) The mean fecal pellet number was significantly higher in dystrophic animals of both strains than in normal animals (mdx: 6.11 ± 0.37 vs. BL10: 0.16 ± 0.07, P = 0.0079, n = 5; mdx52: 2.89 ± 0.33 vs. BL6: 0.00 ± 0.00, P = 0.0022, n = 6). Interestingly, BL6 animals ceased defecation completely during exercise, while normal BL10 animals still had minimal amounts of defecation.

**Fecal output remains constant across all ages regardless of exercise/resting protocol or strain. (A) Resting and (B) exercised fecal output rate over age. (C) Resting and (D) exercised weight per fecal pellet over age. All data are reported as mean values ± SEM with level of significance set at P < 0.05.**
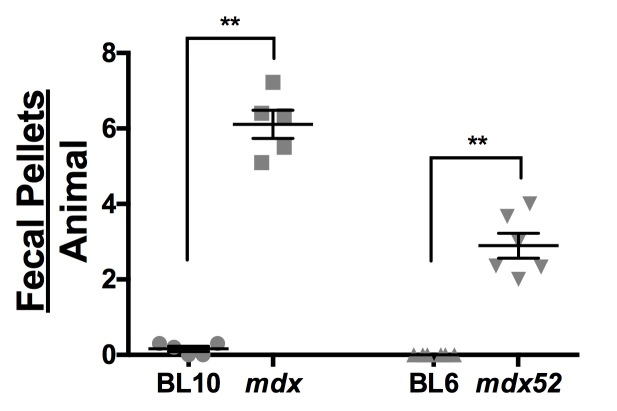

## DISCUSSION

The aim of this study was to further investigate the differences in fecal output of dystrophic (mdx52 and mdx) vs. normal (BL6 & BL10) mice. Therefore, we compared the number of fecal pellets and total fecal weight from dystrophic and normal mice.

Numerous studies have confirmed that a high proportion of DMD patients suffer from gastrointestinal dysfunction such as constipation, gastric hypomotility and delayed gastric emptying 1^, ^3^, ^4^, ^9^, ^10^, ^11^, ^12. This has been linked tentatively to the fatty tissue infiltration, atrophy, fibrosis and interstitial edema in the gastrointestinal tract in the absence of dystrophin within smooth muscles 13.

While our findings of hyperactive gastrointestinal activity in dystrophic mice do not fit straightforwardly with the observations in humans, neither are they contradictory – they may be interpreted as a response to exercise-­induced stress superimposed on a background of fecal retention. This idea is supported by the fact that, in an empty cage, where human handling is the only stress, no difference was noted in fecal output between dystrophic and normal mice. It is intriguing that the BL6 control mice ceased even the low incidence of defecation seen in empty cages when forced to exercise, whereas both dystrophic strains showed distinctly greater fecal output than their normal counterparts in the latter conditions.

Our findings also seem superficially at variance with the reports of Mule et al, of significantly lower fecal pellet output, as well as gastric emptying and longer intestinal transit times in dystrophic mice 5. We suggest that this may reflect the differences between the stresses generated by oral gavage or by the 24­hour food deprivation in the Mule et al. studies, compared with the 20-­minute forced exercise regimen that preceded collection of fecal samples in our study. We suspect that the substantial difference between fecal outputs of exercised dystrophic and normal animals have not been noticed previously because most protocols do not involve exercising the two strains side by side.

As for other tests of behavioral features associated with lack of dystrophin, the basic mechanisms have yet to be elucidated. Morphological changes in the smooth muscle cells of dystrophic mice have been reported, including a reduction in the thickness of intestinal walls 14^, ^15 that might account for their abnormal gastrointestinal functions. However, the disentanglement of neurological from muscular components of what appears to be a stress response is likely to prove challenging.

We also acknowledge the unnatural requirement for mice – who are naturally nocturnal – to perform a forced exercise protocol during the day. While we believe that both exercise and stress increases fecal output, the consistency of exercising the dystrophic and normal mice in the same environmental conditions provides assurance that the difference we observe is significant. If the test were to be employed in an animal facility that used a day-night reversal protocol, it would be important to test whether this has any effect on the outcome.

## CONCLUSIONS

Irrespective of the underlying mechanisms, this study provides evidence that dystrophic mice show alterations in gastrointestinal function that are easily measured by imposition of a low impact procedure. The size and consistency of the difference between dystrophic and normal mice of the two strains we have tested provides a clear and sensitive signal with little or no overlap, and also emphasizes the need to test any given dystrophin mutation against its background strain of origin. We therefore propose the exercise­induced Colo­-Rectal Activation Phenotype (CRAP) test as a simple, non­invasive biomarker that would be useful for serial longitudinal sampling as part of any test to determine the treatment efficacy of therapeutic restoration of dystrophin.

## Corresponding Author

Marie Nearing, MNearing@childrensnational.org and Terence Partridge, TPartridge@childrensnational.org

## Data Availability

All relevant data can be found at Figshare: https://doi.org/10.6084/m9.figshare.5738352.v1.

## Competing Interests

The authors have declared that no competing interests exist.
